# The effect of liraglutide, a GLP-1 analog, on indomethacin-induced gastric ulcers in diabetic rats

**DOI:** 10.1590/acb407325

**Published:** 2025-09-29

**Authors:** Huseyin Emre Arslan, Yasemin Teksen, Orhan Ozatik, Mustafa Cem Algin

**Affiliations:** 1 Kütahya Health Sciences University – Faculty of Medicine – Department of General Surgery – Kutahya – Turkey.; 2 Kütahya Health Sciences University – Faculty of Medicine – Department of Pharmacology – Kutahya – Turkey.; 3 Kütahya Health Sciences University – Faculty of Medicine – Department of Histology and Embryology – Kutahya – Turkey.; 4 Eskisehir City Hospital – Department of General Surgery – Eskisehir – Turkey.

**Keywords:** Stomach Ulcer, Indomethacin, Liraglutide, Glucagon-Like Peptide 1, Rats

## Abstract

**Purpose:**

To investigate the potential pleiotropic effects of liraglutide (LG), a glucagon-like-peptide-1 analog, on gastric ulcer prevention in rats with diabetes induced by streptozotocin (STZ).

**Methods:**

We randomly divided 63 male Wistar rats into seven groups. STZ was administered intraperitoneally (IP) to the animals in the diabetic control (group STZ), diabetic control + indomethacin (INDO) (group STZI), STZ + INDO + omeprazole (group OMP), STZ + INDO + LG (0.2 mg/kg) (group 0.2LG), and STZ + INDO + LG (0.4 mg/kg) group (group 0.4LG). We administered OMP IP to group OMP, 0.2 mg/kg LG to group 0.2LG SC, 0.4 mg/kg LG to group 0.4LG SC, normal saline to non-diabetic control (sham group), group STZ, non-diabetic control + INDO (group KI), and group STZI SC. INDO was administered to the animals in groups KI, STZI, OMP, 0.2LG, and 0.4LG by gavage. Then, the caspase-3, epidermal growth factor (EGF), vascular endothelial growth factor-A (VEGF-A), prostaglandin E2 (PGE2), interleukin-6 (IL-6), tumor necrosis factor-alpha (TNF-α), superoxide dismutase-1 (SOD-1), glutathione (GSH), and malondialdehyde (MDA) levels were studied.

**Results:**

LG prevented INDO-induced ulcers and decreased apoptosis in the stomach tissue. It increased the SOD-1, GSH, EGF, VEGF-A, and PGE2 levels, and reduced the MDA, IL-6, and TNF-α levels. The anti-ulcer effect of LG was lower, but close to that of OMP.

**Conclusion:**

The antioxidant, anti-inflammatory, and anti-apoptotic effects of LG, its ability to regulate EGF, VEGF-A, and PGE2 levels, and its capacity to reduce blood glucose levels in diabetic rats may contribute to its anti-ulcer effect.

## Introduction

Diabetes mellitus (DM) is a metabolic disease characterized by chronic hyperglycemia due to a lack of insulin secretion and decreased insulin efficiency. Hyperglycemia contributes to oxidative stress by causing prolonged neutrophil infiltration and inflammation through the elevation of pro-inflammatory mediators and the generation of reactive oxygen species (ROS) by glucose oxidation. Excessive ROS production leads to the deterioration of antioxidant defenses and metabolic complications^1^.

One of these complications is the deterioration and delay in wound healing. Impaired wound healing and susceptibility to infection due to delayed granulation tissue formation resulting from increased production of advanced glycation products are important clinical problems in patients with diabetes. Angiogenesis facilitates the transport of oxygen, nutrients, and other necessary tools for wound healing to the wound site. In angiogenesis, angiogenic factors such as vascular endothelial growth factor (VEGF) help to form, support, and stabilize new blood vessels. Chronic hyperglycemia in patients with diabetes leads to delayed wound healing by impairing vascular endothelial function, reducing the levels of major angiogenic factors, and impairing angiogenesis^2^^,^ 3.

In DM, wound healing is difficult not only in cutaneous wounds but also in peptic ulcer lesions. A higher incidence of peptic ulcer has been reported in patients with diabetes than in those without it^4–6^. Animal studies have shown increased gastric mucosal sensitivity to ulcerogenic drugs or stress and delayed gastric ulcer healing in diabetic rats^4^. Although clinical and experimental studies have shown that diabetes delays the healing process of peptic ulcers, there are no effective clinical treatment approaches other than anti-peptic ulcer drugs for the treatment of peptic ulcers in diabetic patients.

Glucose-dependent insulinotropic peptide (GIP) and glucagon-like peptide-1 (GLP-1) are two hormones that act as incretins. As the biological half-life of GLP-1 is short (2–3 min), it cannot be used as a drug^7^^,^ 8. In circulation, GLP-1 is degraded by dipeptidyl peptidase IV (DPP-IV). Liraglutide (LG) is a long-acting GLP-1 agonist that is resistant to DPP-IV. LG and other GLP-1 analogs (exenatide, exendin-4, lixisenatide, and dulaglutide) have been approved for blood glucose regulation in DM owing to their insulinotropic effects^9^.

LG has also been reported to be cardioprotective in patients with diabetes by suppressing endothelial cell apoptosis and improving vascular endothelial dysfunction caused by oxidative stress^10^. LG is beneficial in diabetic cardiomyopathy by activating the cytoprotective pathways in rats^11^. LG has been suggested to exert anti-inflammatory, anti-apoptotic, and antioxidant effects^12^. Experimental studies showing the neuroprotective effects of GLP-1 agonists in neurodegenerative diseases such as Alzheimer’s disease, Parkinson’s disease, and stroke are increasing daily^13^. It has also been reported that LG accelerates wound healing and may provide additional benefits in diabetic wounds^14^^,^ 15. Exendin, a GLP-1 analog, has been shown to accelerate peptic ulcer prevention in diabetic rats owing to its anti-oxidative, pro-angiogenic, and anti-inflammatory effects^3^.

Given its anti-inflammatory, antioxidant, anti-apoptotic, and wound-healing efficacies, LG may be beneficial for gastric ulcer development during diabetes. This study investigated the efficacy of LG in an indomethacin-induced gastric ulcer rat model of diabetes.

## Methods

### Diabetic rat model and creation of gastric ulcer

All animal procedures were performed at the Experimental Animal Breeding Research and Application Center and Pharmacology Department Laboratory, according to national and international regulations regarding animal experiments. This study was conducted in accordance with the Animals in Research: Reporting In Vivo Experiments (ARRIVE) guidelines^16^. Permission for this study was obtained from the Animal Experiments Local Ethics Committee (Ethics Committee Decision No. 2019.07.02/08.07.2019).

**Figure 1:**
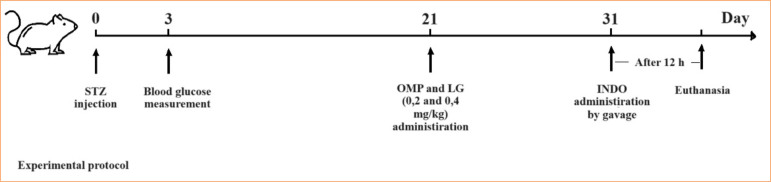
Experimental protocol.

STZ: streptozotocin; OMP: omeprazole; LG: liraglutide; INDO: indomethacin. Source: Elaborated by the authors.

Diabetes was induced in male Wistar-albino rats (weighing approximately 250–300 g) by a single intraperitoneal (IP) injection of streptozotocin (STZ) (65 mg/kg). Only 0.2 mL of citrate buffer was administered intraperitoneally to control animals without diabetes. Three days after the STZ injection, blood glucose levels were measured in blood collected from the tail vein using a glucometer (Lifechek Compakt TD-4283). Animals with a blood glucose level of > 250 mg/dL were considered diabetic. Ulcer model studies were performed three weeks after the STZ injection (Fig. 1).

Three weeks after the STZ injection, LG (Victoza Pen 6 mg/mL, Novo Nordisk) was administered once a day at doses of 0.2 and 0.4 mg/kg subcutaneously (SC) to the nape of the animals for 10 days (the total experimental period was 31 days). LG was diluted with normal saline and administered at an appropriate dose according to the literature^17^. Three weeks after the STZ injection, omeprazole (Omeprol 40 mg vial, Sandoz) was administered IP at the dose of 10 mg/kg once a day for 10 days^18^. The animals in the non-diabetic and STZ control groups were administered SC normal saline (NS) frequently for 10 days.

Indomethacin (INDO), a non-steroidal anti-inflammatory drug (NSAID), is frequently used in experimental gastric ulcer models because it causes gastrointestinal damage, including gastrointestinal bleeding, ulceration, and perforation^19^^,^ 20. Thirty minutes after the last doses of LG and omeprazole (OMP), INDO (Acros) was administered by gavage at the dose of 30 mg/kg (in 0.5 mL of saline)^21^. Twelve hours after the INDO administration, the rats were anesthetized with ketamine (Ketalar, Pfizer) (100 mg/kg IP) + xylazine (10 mg/kg) (Alfazyne), euthanized after intracardiac blood sampling, and their stomachs were removed.

### Treatment protocol

Rats were randomly divided into seven groups (n = 7) as sham (non-diabetic control), STZ (diabetic control), KI (non-diabetic control + INDO), STZI (STZ + INDO), OMP (STZ + INDO + OMP), 0.2LG (STZ + INDO + LG 0.2 mg/kg), and 0.4LG (STZ + INDO + LG 0.4 mg/kg).

The sham, STZ, KI, and STZI groups were administered NS frequently for 10 days starting from day 21. At the end of the experiment, INDO was administered by oral gavage to the KI, STZI, OMP, 0.2LG, and 0.4LG groups. The OMP group was administered OMP IP for 10 days, three weeks after the STZ administration. LG was administered SC frequently for 10 days to the 0.2LG (0.2mg/kg) and 0.4LG (0.4 mg/kg) groups treated with LG.

Body weights and blood glucose levels were recorded on days 3 and 31. On days 31 and 12 h after the INDO administration by gavage, the animals were euthanized, and their blood samples and stomach tissues were collected.

### Measurement of ulcer index

Immediately after euthanasia, the stomach samples were cut along the greater curvature, washed with cold NS, and photographed using a scale. The total area of the gastric mucosa and quantitative analysis of the ulcer area were evaluated using an imaging software (ImageJ Software)^3^.

### Histopathological evaluation

Gastric tissue samples were fixed in 10% buffered neutral formalin. After routine follow-ups, the sections were stained with hematoxylin and eosin (H&E), evaluated under a light microscope (Olympus), and photographed. Lesions observed in the stomach were classified as no lesions (normal stomach) (0), hyperemia (1), hemorrhagic spots (2), mild gastritis (3), moderate gastritis (4), erosive gastritis (5), or severe damage to the mucosa and submucosa (6)^22^.

### Immunohistochemical evaluation of apoptosis

Caspase-3 (cysteine aspartate-specific protease-3) was studied immunohistochemically to detect apoptosis in gastric tissue. After the deparaffinization, the tissues were rehydrated with ethanol, water, and phosphate buffer. The tissues were incubated in an Anti-Caspase-3 (Abcam) solution for 1 h at room temperature. Subsequently, the cells were examined under a light microscope.

### Biochemical analysis of gastric tissue samples

We weighed 0.2 g of stomach tissues and homogenized them with 2 mL of 10% 150 mM phosphate buffer (pH 7.4) in a homogenizer (Benchmark, USA) at 2,000 rpm for 1 min. After the homogenates were centrifuged at 12,000 rpm at +4°C for 10 min (Electo Mag), the supernatants were stored at -80°C until their analysis. The amount of protein in gastric homogenates was determined using a Nano Drop spectrophotometer (Quawell, United States of America).

The epidermal growth factor (EGF) level in gastric homogenates to evaluate wound healing, VEGF-A level in gastric tissues to detect angiogenesis and, thus, ulcer preventing, gastric prostaglandin E2 (PGE2) to evaluate mucosal integrity and ulcer preventing levels, interleukin-6 (IL-6) and tumor necrosis factor-α (TNF-α) levels, which are pro-inflammatory cytokines, to evaluate inflammation in gastric tissue, and the levels of the antioxidant enzymes superoxide dismutase-1 (SOD-1) and glutathione (GSH) in the stomach tissues to evaluate the oxidative stress in gastric ulcer were measured using a rat-specific enzyme-linked immunosorbent adhesion test (ELISA) kit (Elabscience). To evaluate oxidative stress in gastric tissues, the levels of malondialdehyde (MDA), a lipid peroxidation product, were measured colorimetrically using rat kits (Elabscience) and the thiobarbituric acid reactivity method.

### Statistical analysis

The data are presented as the standard error of the mean (SEM). The conformity of continuous variables to normal distribution was evaluated using the Shapiro-Wilk test. One-way analysis of variance (ANOVA) was used for intergroup comparisons of normally distributed variables. Kruskal-Wallis analysis was used to compare non-normally distributed variables between the groups. Statistical significance was set at *p* < 0.05. All analyses were performed using Statistical Package for the Social Sciences (SPSS) 16.

## Results

### Overall results

At the beginning of the experiment, the number of animals in each group was nine. The mortality rates in the diabetic groups treated with STZ were two animals in the STZ control group, two animals in the STZI group, two animals in the OMP group, one animal in the 0.2LG group, and two animals in the 0.4LG group; the number of animals in the experimental groups was rearranged to be seven. Weight gain stopped, and body weight decreased compared to normal 31 days after the STZ administration in the diabetic experimental groups (*p* < 0.001). It was observed that the administration of 0.2 and 0.4 mg/kg LG in the STZ-diabetic rats prevented weight loss at a similar level, and there was no statistical difference between them (Table 1). It was determined that the blood glucose levels of all the STZ-treated groups were statistically significantly higher than those of the control group (*p* < 0.001). The LG treatment decreased the blood glucose levels in a dose-dependent manner. The blood glucose level decreased to 223.71 ± 11.93 mg/dL (*p* < 0.001) in the 0.2 mg/kg LG group, and to 161.57 ± 10.10 mg/dL in the 0.4 mg/kg LG group (*p* < 0.01). It was observed that 0.4 mg/kg LG was more effective in lowering blood glucose levels (*p* < 0.05) (Table 2).

**Table 1:** Effect of liraglutide on body weight in STZ-diabetic rats. Results are presented as mean ± standard error of the mean (n = 7).

Groups	Body weight (g)		% Weight gain
	Day 1	Day 31	
Sham (control non-diabetic)	268.57 ± 5.08	295.71 ± 2.97	10.23 ± 1.26
STZ (control diabetic)	270.00 ± 5.34	252.86 ± 5.22	-6.2 ± 2.25**
KI	265.71 ± 4.29	288.57 ± 2.61	8.72 ± 1.50
STZI	277.14 ± 3.60	260.00 ± 3.78	-6.13 ± 1.5**
OMP	267.14 ± 2.86	251.43 ± 4.04	-5.80 ± 2.0**
0.2LG	268.57 ± 2.61	270.00 ± 2.18	0.57 ± 1.0** ^
0.4LG	270.00 ± 3.09	277.14 ± 2.86	2.73 ± 1.6* #

STZ: streptozotocin; KI: non-diabetic control + indomethacin; STZI: streptozotocin + indomethacin; OMP: omeprazole; LG: liraglutide;

* *p* < 0.01;

** *p* < 0.001 (*versus* sham);

^ *p* < 0.01;

# *p* < 0.001 (versus STZ control diabetic).

Source: Elaborated by the authors.

**Table 2:** Effect of liraglutide on blood glucose level in STZ-diabetic rats. Results are presented as mean ± standard error of the mean (n = 7).

Groups	Blood glucose level (mg/dL)	
	Day 3	Day 31
Sham (control non-diabetic)	79.28 ± 5.92	82.14 ± 5.22
STZ (control diabetic)	379.29 ± 9.97	387.14 ± 10.68**
KI	83.71 ± 5.14	89.43 ± 3.15
STZI	358.57 ± 13.66	375.71 ± 12.74**
OMP	349.29 ± 9.09	357.86 ± 10.40**
0.2LG	369.29 ± 8.66	223.71 ± 11.93** ^
0.4LG	391.00 ± 6.73	161.57 ± 10.10* ^ +

STZ: streptozotocin; KI: non-diabetic control + indomethacin; STZI: streptozotocin + indomethacin; OMP: omeprazole; LG: liraglutide;

* *p* < 0.01;

** *p* < 0.001 (*versus* control non-diabetic);

^ *p* < 0.01 (*versus* STZ control diabetic);

+ *p* < 0.05 (*versus* 0.2LG).

Source: Elaborated by the authors.

### Liraglutide shows peptic ulcer prevention in diabetic rats

**Figure 2:**
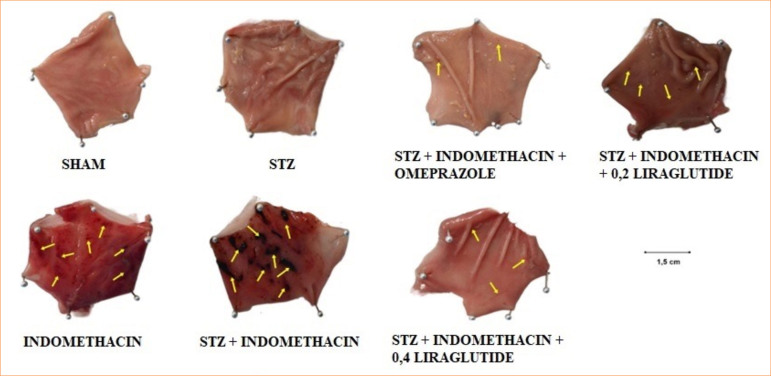
Ulcerated areas in the stomach in the experimental groups.

**Figure 3:**
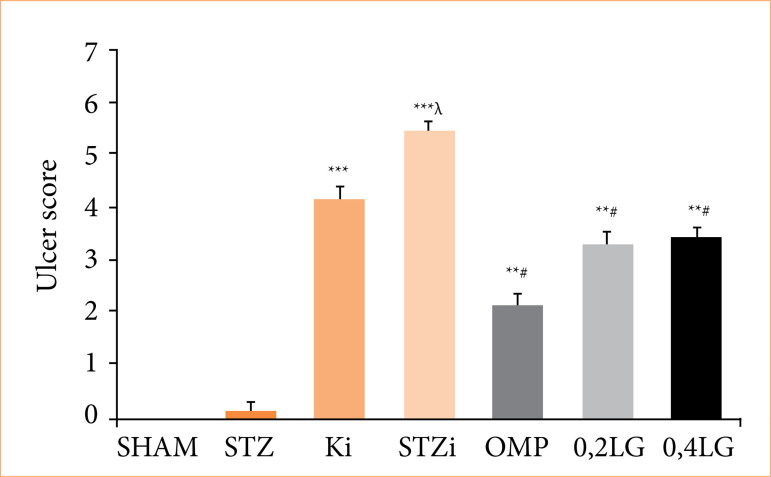
Effect of liraglutide on histopathological ulcer score in STZ diabetic rats. Results are presented as mean ± standard error of the mean (n = 7). Analysis of variance.

STZ: streptozotocin; KI: non-diabetic control + indomethacin; STZI: streptozotocin + indomethacin; OMP: omeprazole; LG: liraglutide (0.2 and 0.4 mg/kg); **p < 0.01; ***p < 0.001 (*versus* sham and STZ control diabetic); λ*p* < 0.001 (*versus* all groups); #*p* < 0.05 (*versus* STZI). Source: Elaborated by the authors.

No gastric ulcers were found in the sham and STZ diabetic groups that did not receive INDO. In the non-diabetic control group, the ulcer index increased (4.27 ± 0.28 mm^2^), and significant ulceration was observed (*p* < 0.001 compared to the sham group) with the administration of INDO. The ulcer index was higher in the STZ diabetic group than in the non-diabetic group (6.43 ± 0.42 mm2) (*p* < 0.001), and diabetes increased ulcer formation (*p* < 0.05 compared to the KI group). The OMP administration to STZ diabetic rats for 10 days before INDO significantly reduced the ulcer index (1.31 ± 0.08 mm^2^) (*p* < 0.001, different from all the groups). It was determined that the ulcer index of the 0.2 mg/kg LG group decreased compared to the STZ diabetic group (4.52 ± 0.18 mm^2^) (*p* < 0.05), but there was no difference between the 0.2 mg/kg LG group and the non-diabetic group. In the group that received 0.4 mg/kg LG, it was determined that the ulcer index decreased significantly compared to the STZ group (3.97 ± 0.26 mm^2^) (*p* < 0.05), but it was similarly effective in preventing ulcers compared to the 0.2 mg/kg dose. Both doses of LG reduced the ulcer index to a lesser extent than the OMP (Figs. 2 and 3).

STZ: streptozotocin. Source: Elaborated by the authors.

### Histopathological evaluation

**Figure 4:**
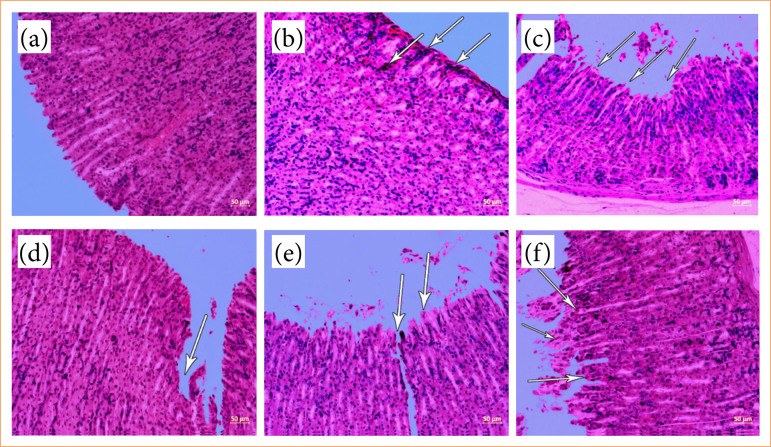
Histopathological examination of stomach tissue samples (x20). **(a)** In the STZ control diabetic group, the stomach is observed in a normal histological structure with all its layers and cells. **(b)** In the non-diabetic group administered indomethacin, moderate gastric gastritis, erosive gastritis, and lesions up to severe damage to the mucosa and submucosa were observed. **(c)** Severe damage, gastritis, and hemorrhage were detected in the gastric mucosa and submucosa in the STZ diabetic group administered indomethacin. **(d)** In the STZ diabetic group administered omeprazole, the damage to the gastric mucosa and submucosa decreased, some animals had weak gastritis findings, mild hyperemia, and hemorrhage. **(e)** Although ulcer findings were reduced in the STZ diabetic group treated with 0.2 mg/kg liraglutide, healing was less frequent than in the groups given omeprazole. **(f)** Ulcer findings decreased in the STZ diabetic group administered 0.4 mg/kg liraglutide, and the healing was not different from the group administered 0.2 mg/kg liraglutide.

The histopathological examination of the preparations obtained by H&E staining revealed normal histological findings in the sham group and STZ diabetic groups (Fig. 4a). Moderate gastritis, erosive gastritis, and pathological changes leading to severe damage to the mucosa and submucosa were detected in the INDO-treated control group (Fig. 4b). In STZ diabetic rats administered INDO, severe damage to the mucosa and submucosa, gastritis, and hemorrhage were observed (Fig. 4c). It was observed that damage to the mucosa and submucosa decreased in the group treated with OMP; weak gastritis findings remained in some animals, and they had mild hyperemia and hemorrhage (Fig 4d). Although the ulcer findings were reduced in the LG-administered groups, prevention was less common than in the OMP-treated groups (Figs. 4e and 4f). Ulcer prevention was similar in the 0.2 and 0.4 mg/kg LG groups, and there was no dose-dependent effect (Fig. 4).

STZ: streptozotocin. Source: Elaborated by the authors.

### Liraglutide reduces apoptosis in diabetic rats

**Figure 5:**
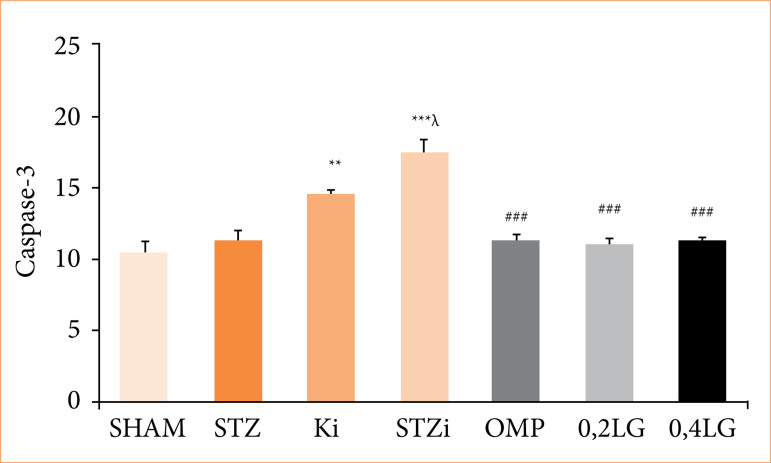
Effect of liraglutide on apoptosis in STZ diabetic rats. Results are presented as mean ± standard error of the mean (n = 7). Analysis of variance.

STZ: streptozotocin; KI: non-diabetic control + indomethacin; STZI: streptozotocin + indomethacin; OMP: omeprazole; LG: liraglutide (0.2 and 0.4 mg/kg); **p < 0.01; ***p < 0.001 (versus sham and STZ control diabetic); λ*p* < 0.001 (versus all groups); ###*p* < 0.001 (versus STZI). Source: Elaborated by the authors.

**Figure 6:**
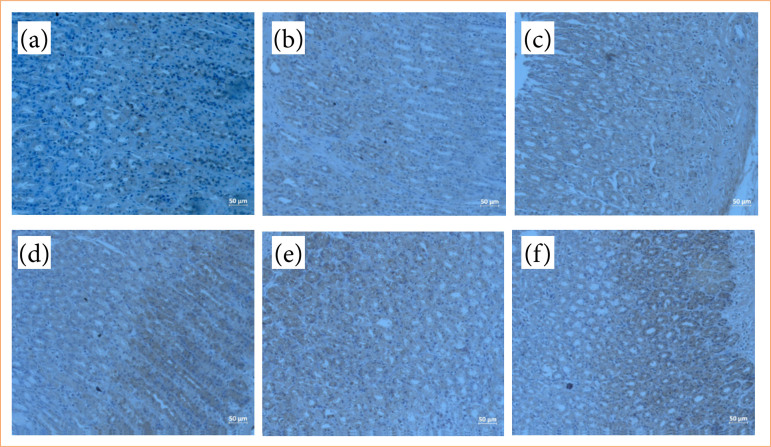
Caspase-3 immunohistochemical staining of stomach tissue samples (x20). **(a)** Caspase-3 immunohistochemical staining of gastric tissue in STZ control diabetic group. **(b)** Increased caspase-3 immunohistochemical staining in gastric tissue in the non-diabetic group treated with indomethacin. **(c)** Increased caspase-3 immunohistochemical staining in gastric tissue in STZ diabetic group treated with indomethacin. **(d)** Decreased caspase-3 immunohistochemical staining in gastric tissue in STZ diabetic group treated with omeprazole. **(e)** Decreased caspase-3 immunohistochemical staining in gastric tissue in STZ diabetic group administered 0.2 mg/kg liraglutide. **(f)** Decreased caspase-3 immunohistochemical staining in gastric tissue in STZ diabetic group administered 0.4 mg/kg liraglutide.

Caspase-3 was studied immunohistochemically to detect apoptosis in the gastric tissue (Fig. 5). Apoptosis increased in the control and STZ diabetic groups treated with INDO (*p* < 0.01 compared to the sham group) (Figs. 6a, 6b, and 6c), but decreased significantly in the groups treated with OMP and LG, and there was no dose-dependent effect (p < 0.001 compared to the STZI group) (Figs. 6d, 6e, and 6f).

STZ: streptozotocin. Source: Elaborated by the authors.

### Liraglutide’s effects on angiogenesis and mucosal integrity in diabetic rats

**Figure 7:**
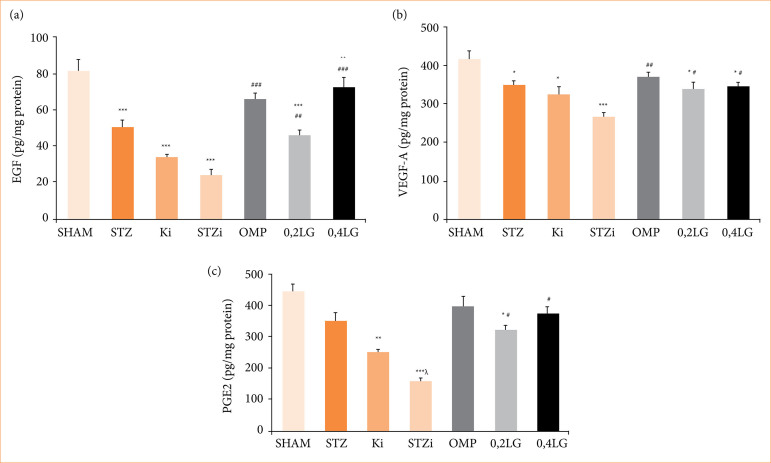
Effect of liraglutide on ulcer healing. **(a)** Effect of liraglutide on EGF levels in gastric tissue of STZ diabetic rats. **(b)** Effect of liraglutide on VEGF-A levels in gastric tissue in STZ diabetic rats. **(c)** Effect of liraglutide on PGE2 levels in gastric tissue of STZ diabetic rats. Results are presented as mean ± standard error of the mean (n = 7). Analysis of variance.

STZ: streptozotocin; KI: non-diabetic control + indomethacin; STZI: streptozotocin + indomethacin; OMP: omeprazole; LG: liraglutide (0.2 and 0.4 mg/kg); EGF: epidermal growth factor; VEGF-A: vascular endothelial growth factor-A; PGE2: prostaglandin E2; *p < 0.05, **p < 0.01 and ***p < 0.001 (*versus* sham); ^#^ *p* < 0.05, ^##^ *p* < 0.01 and ^###^ *p* < 0.001 (*versus* STZI); ^^*p* < 0.01 (versus 0.2 LG); λ*p* < 0.001 (*versus* all groups). Source: Elaborated by the authors.

The EGF, VEGF-A, and PGE2 levels were measured in gastric homogenates to evaluate angiogenesis, mucosal integrity, and ulcer prevention. The EGF and VEGF-A levels decreased in the gastric tissue of the STZ diabetic group (*p* < 0.05 compared to the sham group). The PGE2 levels also decreased, but the difference was not statistically significant. The administration of INDO to the non-diabetic control group significantly decreased the levels of EGF, VEGF-A, and PGE2 (*p* < 0.05 compared to the sham group). The lowest EGF and VEGF-A levels were observed in the STZ diabetic group treated with INDO; however, no statistically significant difference was observed in the KI group. The lowest PGE2 level was observed in the STZ diabetic group treated with INDO (p < 0.05). LG increased the EGF levels in a dose-dependent manner, while the VEGF-A and PGE2 levels were independent of the dose. It was determined that the VEGF-A levels increased significantly compared to the STZI and KI groups, but did not reach the levels in the sham group (*p* < 0.05). The EGF level was measured as 45.94 ± 5.82 pg/mg protein in the 0.2 mg/kg LG group, and 72.07 ± 3.17 pg/mg protein in the 0.4 mg/kg LG group. It was observed that 0.4 mg/kg LG was more effective in increasing the EGF levels (*p* < 0.01 compared with the 0.2 LG group). The PGE2 level was measured as 325.08 ± 14.44 pg/mg protein in the 0.2 mg/kg LG group, and 377.63 ± 26.00 pg/mg protein in the 0.4 mg/kg LG group, and no difference was observed between the groups. Both doses of LG increased the EGF, PGE2, and VEGF-A levels at a rate similar to that of OMP (Figs. 7a, 7b, and 7c).

### Liraglutide reduces inflammation in gastric ulcer

**Figure 8:**
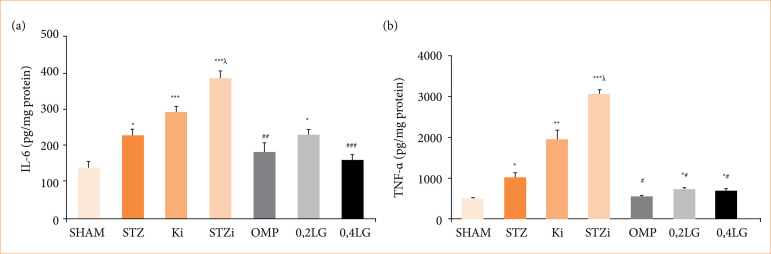
Effect of liraglutide on inflammation. **(a)** Effect of liraglutide on IL-6 levels in gastric tissue of STZ diabetic rats. **(b)** Effect of liraglutide on TNF-α levels in gastric tissue of STZ diabetic rats. Results are presented as mean ± standard error of the mean (n = 7). Analysis of variance.

STZ: streptozotocin; KI: non-diabetic control + indomethacin; STZI: streptozotocin + indomethacin; OMP: omeprazole; LG: liraglutide (0.2 and 0.4 mg/kg); IL-6: interleukin-6; TNF: tumor necrosis factor; *p < 0.05, **p < 0.01 and ***p < 0.001 (versus sham); ^#^ *p* < 0.05 (versus STZI); ^##^ *p* < 0.01 and ^###^ *p* < 0.001 (*versus* KI); ^^*p* < 0.01 (*versus* 0.2 LG); λ*p* < 0.001 (*versus* all groups). Source: Elaborated by the authors.

Regional inflammation in gastric tissue was determined by proinflammatory cytokines IL-6 and TNF-α. The TNF-α and IL-6 levels were higher in the gastric tissue in the STZ diabetic group (p < 0.05 compared to the sham group). In the non-diabetic control group, INDO administration significantly increased the IL-6 and TNF-α levels (*p* < 0.01, compared to the sham group). The highest TNF-α and IL-6 levels were observed in the STZ diabetic group treated with INDO (*p* < 0.05). LG reduced the TNF-α and IL-6 levels in the stomach tissues. The TNF-α levels were measured as 707.32 ± 51.38 pg/mg protein, and the IL-6 levels as 223.64 ± 16.01 pg/mg protein in the 0.2 mg/kg LG group. The TNF-α levels were measured as 684.05 ± 43.14 pg/mg protein, and the IL-6 levels were 157.45 ± 17.87 pg/mg protein in the 0.4 mg/kg LG group. Both doses of LG reduced the TNF-α level at a similar rate to OMP, and no difference was observed between the groups. Although the IL-6 levels were lower in the 0.4 mg/kg LG group, there was no statistical difference between the two doses (Figs. 8a and 8b).

### Liraglutide reduces oxidative stress in gastric ulcer

**Figure 9:**
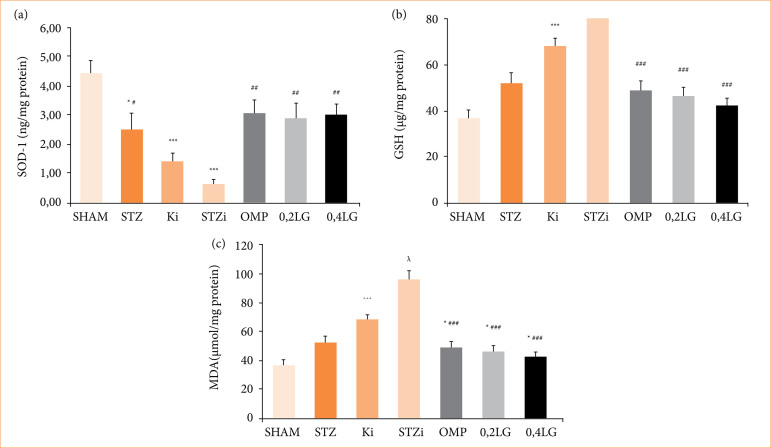
Effect of liraglutide on oxidative stress. **(a)** Effect of liraglutide on SOD-1 levels in gastric tissue of STZ diabetic rats. **(b)** Effect of liraglutide on GSH levels in gastric tissue of STZ diabetic rats. **(c)** Effect of liraglutide on MDA levels in gastric tissue of STZ diabetic rats. Results are presented as mean ± standard error of the mean (n = 7). Analysis of variance.

STZ: streptozotocin; KI: non-diabetic control + indomethacin; STZI: streptozotocin + indomethacin; OMP: omeprazole; LG: liraglutide (0.2 and 0.4 mg/kg); SOD-1: superoxide dismutase 1; GSH: glutathione; MDA: malondialdehyde; *p < 0.05, **p < 0.01 and ***p < 0.001 (versus sham and STZ); ^#^ *p* < 0.05 (*versus* STZI); ^##^ *p* < 0.01 and ^###^ *p* < 0.001 (*versus* STZI); ^^*p* < 0.01 (*versus* 0.2 LG); λ*p* < 0.001 (*versus* all groups). Source: Elaborated by the authors.

Oxidative stress in gastric ulcers was determined by measuring the levels of SOD-1 and GSH, which are antioxidant-acting enzymes in gastric tissues, and MDA, which is a lipid peroxidation product. In the STZ diabetic group, the level of SOD-1 in the stomach tissue decreased (*p* < 0.05 compared to the sham group), and the GSH and MDA levels were similar to those in the sham group. In the non-diabetic control group, INDO administration significantly decreased the SOD-1 and GSH levels and significantly increased the MDA levels (*p* < 0.001 compared with the sham and STZ groups). The lowest SOD-1 and GSH levels and the highest MDA levels were observed in the STZ diabetic group treated with INDO (*p* < 0.001). LG increased the levels of SOD-1 and GSH and decreased the level of MDA in the stomach tissue. In the 0.2 mg/kg LG group, the SOD-1, GSH, and MDA levels were measured as 2.90 ± 0.52 ng/mg protein, 40.81 ± 2.30 µg/mg protein, and 46.50 ± 3.96 µmol/mg, respectively. In the 0.4 mg/kg LG group, the SOD-1, GSH, and MDA levels were measured as 3.04 ± 0.37 ng/mg protein, 43.80 ± 2.86 µg/mg protein, and 42.25 ± 3.68 µmol/mg protein, respectively. No differences were observed between the groups. Both doses of LG increased the SOD-1 and GSH levels at a rate similar to that of OMP and decreased the MDA levels at a rate similar to that of OMP (Figs. 9a, 9b, and 9c).

## Discussion

In our animal experiment study, the administration of 0.2 and 0.4 mg/kg LG was compared with the OMP and control groups in terms of ulcer index and score, ulcer prevention, oxidative stress, inflammation, and apoptosis in the rats with gastric ulcer formed with INDO. The 0.2 and 0.4 mg/kg LG treatments prevented ulcer formation with antioxidant, anti-inflammatory, and anti-apoptotic effects. The antiulcer effect of LG was not dose-dependent and was lower than that of OMP. There was a significant difference in terms of the variables compared to the control group.

This is the first study to investigate the effects of LG on acute gastric ulcers. To the best of our knowledge, only one animal experimental study has evaluated the effect of exendin-4, a GLP-1 analog similar to LG, on chronic ulcers induced by the submucosal injection of acetic acid into the stomachs of rats. In the present study, exendin-4 was shown to accelerate ulcer healing^3^. In our study, LG prevents ulcer formation at both the 0.2 and 0.4 mg/kg doses, both at macroscopic and microscopic levels (ulcer index, ulcer score). This positive effect on ulcer prevention may be due to the anti-apoptotic, anti-inflammatory, and antioxidant properties of LG.

Both clinical and animal experimental studies have evaluated the effects of LG on inflammation; however, no study has investigated its effect on regional inflammation in the gastric tissue. Luo et al.^23^, in their study investigating the efficacy of LG in a non-alcoholic fatty liver disease (NAFLD) model in diabetic mice, reported that the levels of TNF-α, IL-1β, and IL-6 were high in the NAFLD group and that the levels of inflammatory cytokines decreased, and hepatic steatosis regressed with LG.

In their study investigating the effect of LG on pancreatic islet grafts *in vitro*, Langlois et al.^24^ reported the anti-inflammatory and antioxidant properties of LG and its beneficial effects on islet graft survival.

Animal studies report that the delayed healing of peptic ulcer in diabetic rats is caused by increased pro-inflammatory reactions (TNF-α, IL-1β, IL-6, and myeloperoxidase) and decreased regenerative activity (MMP-2, IL-10, and cyclic adenosine monophosphate)^3^. In their study on the effects of exendin-4, a GLP-1 analog, on gastric ulcer healing in a gastric ulcer model of diabetic rats, Chen et al.3 reported that the IL-1β and IL-6 levels increased, the IL-10 levels decreased in the gastric tissue of diabetic rats, and the IL-1β, IL-6, and IL-10 levels approached control group levels with the exendin-4 treatment^3^.

In our study, regional inflammation in the stomach tissue was determined by the proinflammatory cytokines IL-6 and TNF-α. LG reduced the TNF-α and IL-6 levels in the stomach tissues. Both doses of LG reduced the TNF-α level at a similar rate to OMP, and no difference was observed between the groups. Although the IL-6 levels were lower in the 0.4 mg/kg LG group, there was no statistical difference between the two doses.

Angiogenic growth factors like EGF, PGE2, and VEGF-A are among the factors that play a role in the reconstruction of the gastrointestinal mucosa, mucosal defense, and ulcer preventing^25^^,^ 26. Chairmandurai et al.^27^ reported that the administration of recombinant human EGF (rhEGF) can alleviate or heal antral ulcers in a gastric ulcer model created with NSAID^27^. Increased EGF levels may improve wound healing by promoting granulation tissue formation in diabetic wounds and ulcers^28–30^. In addition, PGE2 accelerates the healing of peptic ulcers and prevents ulcer formation by stimulating angiogenesis through the upregulation of VEGF-A expression and activation of EP4 (prostaglandin E receptor) receptors^25^. In this study, the EGF, VEGF-A, and PGE2 levels were measured in gastric homogenates to evaluate angiogenesis, mucosal integrity, and ulcer prevention. LG increased the EGF levels in a dose-dependent manner, while the VEGF-A and PGE2 levels were independent of the dose. Both doses of LG increased the EGF, PGE2, and VEGF-A levels at a rate similar to that of OMP. These effects of LG supported its ability to enhance angiogenesis and mucosal integrity in patients with diabetic gastric ulcers.

Oxidative stress induces apoptosis in diabetic rats by activating various caspases as a result of the translocation of cytochrome C from the mitochondria, resulting in DNA damage. Increased ROS generation triggers apoptosis and programmed cell death^31^^–^ 34. Studies on INDO-induced peptic ulcers and jejunal mucosal damage have revealed increased caspase-3 levels^35^^–^ 38. Previous studies have shown a decrease in antioxidant levels (*e.g.*, GSH and SOD) and an increase in MDA levels in the gastric mucosa of rats administered INDO^39^^,^ 40. STZ administration to experimental animals increased MDA and decreased the catalase, GSH-Px, and SOD antioxidant enzyme levels^41^^–^ 46. In our study, the administration of STZ and INDO to experimental animals induced oxidative stress and apoptosis. To detect apoptosis in gastric tissue, caspase-3 was studied immunohistochemically. To detect oxidative stress, SOD-1, and GSH, which are antioxidant enzymes, and MDA, a lipid peroxidation product, were studied. When LG and OMP were administered to STZ diabetic experimental animals treated with INDO, apoptosis decreased in the OMP and LG groups, and the levels of SOD-1 and GSH, which are antioxidant-effective enzymes, increased, as the levels of MDA decreased. The effect of LG on apoptosis and oxidative stress markers was dose-independent and similar to that of OMP.

This study has some limitations that must be pointed out. First, in our study we evaluated the effects of LG on indomethacin-induced acute gastric ulcer, an NSAID. However, there are many factors that cause peptic ulcer, including excessive acid secretion, cortisone-based therapies, alcohol, smoking, stress, heredity, other comorbidities, Helicobacter pylori, and viruses. The effects of LG on peptic ulcer due to these factors should be evaluated in appropriate animal models. Especially the evaluation of the effects of LG on chronic gastric ulcer with acetic acid- and acetic acid + Helicobacter pylori-induced chronic gastric ulcer animal models will strengthen our results.

Secondly, as delayed wound healing due to diabetes is closely related to the duration of diabetes, it is important to evaluate the effects of LG in STZ-diabetic animals for longer than four weeks, for example, 12 or 24 weeks. Thirdly, LG dose-dependently lowered blood glucose, but its protective effect in peptic ulcer was similar at both doses. This may be because LG is given for a short period of time. On the other hand, the beneficial effects of LG on peptic ulcer may be independent of the hypoglycemic effect or direct neutralization of gastric acid. LG probably promotes tissue granulation and ulcerative wound closure by promoting pro-angiogenic response, increasing EGF and PGE2, suppressing regional inflammation, apoptosis, and oxidative stress in ulcerated tissue. Fourthly, mortality is common in the STZ diabetic model. In our study, the number of animals in each group was reduced to seven due to mortality. The use of larger numbers of animals in future studies will strengthen the results.

## Conclusion

LG showed a protective effect against peptic ulcer in diabetic rats, and this effect was lower than OMP in terms of ulcer index, and histopathology. The beneficial effects of LG on peptic ulcer may be independent of the hypoglycemic effect or direct neutralization of gastric acid. Liraglutide probably promotes tissue granulation and ulcerative wound closure by promoting pro-angiogenic response, increasing EGF and PGE2, suppressing regional inflammation, apoptosis and oxidative stress in ulcerated tissue. These results support the clinical use of GLP-1R analogues as adjunctive hypoglycemic agents in the treatment of peptic ulcer disease. As LG has not been tested in diabetic patients with peptic ulcers, the results of this study may serve as a precursor for future clinical trials investigating the effects of LG on ulcer prevention.

## Data Availability

The data supporting the findings of this study are available from the corresponding author upon reasonable request.
